# Plant-Made Biologics

**DOI:** 10.1155/2014/418064

**Published:** 2014-06-02

**Authors:** Qiang Chen, Luca Santi, Chenming Zhang

**Affiliations:** ^1^The Biodesign Institute, Arizona State University, 1001 S. McAllister Avenue, Tempe, AZ 85287, USA; ^2^School of Life Sciences, Arizona State University, 1001 S. McAllister Avenue, Tempe, AZ 85287, USA; ^3^Department of Science and Technology for Agriculture, Forestry, Nature and Energy (DAFNE), University of Tuscia, Via San Camillo de Lellis snc, 01100 Viterbo, Italy; ^4^Department of Biological Systems Engineering, Virginia Tech, Blacksburg, Virginia, VA 24061, USA

The increasing world demand for human biologics cannot be met by current production platforms based primarily on mammalian cell culture due to prohibitive cost and limited scalability [1]. Recent progress in plant expression vector development, downstream processing, and glycoengineering has established plants as a superior alternative to biologic production [2–4]. Plants not only offer the traditional advantages of proper eukaryotic protein modification, potential low cost, high scalability, and increased safety but also allow the production of biologics at unprecedented speed to control potential pandemics or with specific glycoforms for better efficacy or safety (biobetters) [5, 6]. The approval of the first plant-made biologic (PMB) by the United States Food and Drug Administration (FDA) for treating Gaucher's disease heralds a new era for PMBs and sparks new innovations in this field [7, 8].

This special issue aims to showcase the recent developments and application of PMBs in areas of plant host systems, expression vectors, novel vaccine candidates, glycoengineering and posttranslational modification, and economic impact and evaluation. Eight original research and review articles among submissions are selected for this special issue.

Manufacturing costs are a prime determinant of the market acceptability, availability, and profitability of the product for its manufacturer. One of the potential traditional advantages of plant-based systems is their ability to lower the production cost of recombinant biologics. Lower manufacturing costs have been widely assumed as an innate feature of plant-based production platforms because they forego the need for capital investments to build sophisticated cell culture facilities and expensive culture media for biomass generation. However, information on the actual costs of producing PMB at industrial scale is not readily available and reports of serious studies in this area are scarce in the scientific literature. Thus, accurately documenting such an advantage is crucial for plant-based systems to be recognized as a serious platform for manufacturing protein biologics. Tusé et al. provided such an important study in this issue. They reported two case studies on plant-made enzymes. One focused on human butyrylcholinesterase (BuChE) produced in greenhouse-cultivated* Nicotiana* plants for use as a medical countermeasure and the other on cellulases produced with plants grown in the field for ethanol production as a fuel extender. Using reported data and SuperPro Designer modeling software, the authors examined process unit operations and estimated the bulk active product and per-dose or per-unit costs. Their analyses demonstrate that a plant-based platform can substantially reduce the cost of these enzymes compared with traditional platforms. For example, the unit production costs for the plant-made BuChE are calculated to be approximately $234 or $474 per dose, respectively, dependent on whether or not facility dependent costs are included in the estimation. This is in stark contrast to the ~$10,000/dose production cost estimated for blood-derived BuChE. Similarly, the study concludes that for the cellulase enzyme, using the plant-based system may result in a >30% reduction in unit production costs and an 85% reduction in the required capital investment compared with the current fungal-based system. The authors did caution that the cost advantages of PMBs are molecule/product-specific and dependent upon the cost improvement of alternative production platforms. This report presents case studies of PMBs for diverse applications and provides urgently needed technoeconomic evaluations of the current PMB platform.

Two of the original research papers report on the development of plant-made vaccines against infectious diseases. He et al. report their findings of using the domain III (DIII) of West Nile virus (WNV) envelope protein as a vaccine candidate for WNV. They found that* N. benthamiana* plants could produce this antigen efficiently. They also showed the advantage of plant-derived DIII in downstream processing; unlike the insoluble WNV DIII produced in* E. coli*, plant-derived DIII is soluble and readily enriched to high purity without the need for denaturing and refolding. Furthermore, plant-produced DIII was shown to evoke a potent DIII-specific humoral response in mice. No vaccine against WNV is currently available for human use and this study presents an effort towards developing efficacious vaccines against this virus. Another paper in this category investigated the possibility of developing a plant-based vaccine against norovirus Narita 104 (Na) using virus-like particles (VLPs) assembled from the capsid protein (NaVCP). The results showed that expression of NaVCP caused severe leaf necrosis that limited its accumulation in plants. However, plant-produced VLPs were observed by microscopy and induced mucosal and serum antibody responses in mice when delivered intranasally. The authors proposed that Narita 104 VLPs could be a component of a multivalent subunit vaccine.

Downstream processing of target proteins represents a major cost for the overall cost of goods in PMB production. Therefore, reducing cost of PMB extraction and purification will facilitate the commercialization of plant-based production platform and products. A paper in this issue reports an alternative way to extract protein from plants. Instead of homogenization of plant tissue, the authors used a technique called vacuum infiltration-centrifugation (VI-C) to isolate recombinant proteins that are targeted for secretion. Their results indicated that three rounds of VI-C recovered 97% of the secreted proteins accessible to the procedure. While the VI-C procedure was successful for a truncated E1 endoglucanase, the full length E1 enzyme was not recovered as efficiently by the same procedure, indicating the method's limitation on the size of target proteins or the need for technical optimization. However, this study does represent an alternative downstream process for recovering secreted proteins from plant tissue (apoplast) that can potentially drastically reduce the production cost.

Several review articles are also included in this special issue. Two of them discuss the new plant hosts and expression strategies for PMB production and compare them with other alternative manufacturing systems. Hudson et al. report the use of soybean seeds for the expression of a nontoxic form of* S. aureus* enterotoxin B (mSEB). As a natural protein source, soybean seeds allow for an extended storage time under ambient conditions and, thus, can facilitate a more flexible processing schedule. The study results demonstrated an impressive production of ~76 theoretical doses of human vaccine per single soybean seed. Merlin et al. present a comprehensive review of different production strategies applied to four well-characterized, yet very diverse PMBs. The authors emphasize that plant-based production platforms represent a whole array of different strategies that need to be carefully evaluated, in terms of not only mere product yield, but also product quality, production scalability, costs, and cGMP compliance. For a given PMB, the optimal pairing of a plant production host with the most appropriate expression and/or downstream processing strategy often determines its ultimate success. The four case studies center on four different classes of biologics: (i) human glutamic acid decarboxylase (hGAD65), a promising candidate for treating autoimmune type 1 diabetes, (ii) Norwalk VLPs assembled from the Norwalk virus coat protein VP1 for vaccine development, (iii) monoclonal antibody (mAb) 2G12, an anti-HIV-1 human IgG1, a potential human therapeutics against HIV, and (iv) human interleukin-6 (hIL-6), a secreted glycoprotein belonging to the cytokine family. Production of these biologics with various plant systems and expression strategies is examined. The plant systems range from leaf-based production (tobacco,* N. benthamiana*, lettuce, and* Arabidopsis*) to seeds (tobacco,* Arabidopsis*, maize, and petunia), fruits (tomato), and tubers (potato), with both stable and transient expression approaches. Moreover, the production of the fourth-mentioned biologics in plant systems is compared with that of traditional fermenter-based systems such as* E. coli*, yeast, mammalian, and insect cells. The authors highlight the advantages of plant-based systems over fermenters, particularly for certain niche markets. They conclude that plant-based platforms are most beneficial for the production of biologics that require high quantity, rapid production speed, complex post-translational modifications, or oral delivery.

Recent vector development for PMBs is also discussed. Recognizing the potential pitfalls of recombinant protein production in stably transformed plants, a concise review on the novel transient expression systems based on the use of plant virus expression vectors is presented. The author concludes that transient expression systems can overcome the challenges associated with systems based on transgenic plants such as low protein accumulation and long development time, and they can reduce the potential risk of transgene spread from transgenic plants to other plants in the environment and thus alleviate the public concerns.

Overall, the papers in this special issue highlight the recent progress in the PMB field. It is our hope that these papers will provide pertinent information for not only the PMB community, but also the overall scientific and business community for the further consideration and acceptance of plant-based systems as a viable platform for the development and manufacture of human biologics.
